# Mental health survival kit

**DOI:** 10.1590/1516-3180.b2021.139120102020

**Published:** 2020-10-26

**Authors:** Guido Arturo Palomba

**Affiliations:** I Forensic Psychiatrist and Emeritus Member, Academia de Medicina de São Paulo, São Paulo (SP), Brazil.

The book “Mental health survival kit”, written by the illustrious Danish physician Peter Gøtzsche, ought to be obligatory reading material for all who wish to specialize in psychiatry or, indeed, all who already are psychiatrists. The evidence that it provides unquestionably shows the degree of decadence into which this specialty has now fallen.^1^ This book not only warns about the problem but also provides recommendations.

Starting in the second half of the 1980s, psychiatry came to be completely eaten up by the pharmaceutical industry. Through well-designed marketing, the industry implanted a pandemic of medications into the mentality of psychiatry, especially with regard to antidepressants.

Peter Gøtzsche draws attention to some extraordinarily important facts that have emerged through serious meta-analyses that involved several countries. For example, he points out that use of antidepressants significantly increases the number of suicides. Moreover, he shows that great difficulty exists in publishing articles critical of psychotropic drugs, given that this would go against the interests of multibillion-dollar pharmaceutical companies that, by the way, form part of all the main stock markets around the world.

Another significant fact that merits reflection among all psychiatrists is that, beyond the issue of prescription of antidepressants, especially when used for long periods, withdrawal from their use is difficult. Antidepressants cause physical dependence, and withdrawal leads to abstinence syndrome and severe effects on users' mental and physical health. This is not just the author's opinion based on his authority as an internationally respected physician, but is based on scientific evidence that is available for anyone who wishes to see it. It comes from statistical studies that bring together the results from several independent trials conducted in different countries, with systematic reviews of the literature, which obviously minimizes the chances of error.

It should be recalled that Gøtzsche was one of the founders of the Cochrane Collaboration, in Oxford, together with several other eminent physicians of international renown. Among these was the Brazilian Álvaro Nagib Atallah, who introduced evidence-based medicine here in Brazil. This teaches a way of seeing and practicing medicine that, incidentally, forms the necessary safe path that we doctors should follow.

The book proposes solutions that are far from easy. It shows that the current generation of psychiatrists is lost, a view that I fully agree with. At this point, the situation can no longer be reversed, such is the magnitude of the contamination of the minds of today's psychiatrists, who think that the human being is only or almost only a pile of neurons and neurotransmitters that needs drugs that they prescribe.

No, it is not at all like this. On the contrary, these medications are no more than a chemical straitjacket that acts on a biopsychosocial being, to tie it up inside itself. Moreover, these medications give rise to important side effects. This lost generation of psychiatrists can be seen to be negligent, imprudent and lacking in expertise regarding this extremely serious problem. To give a faint idea of the low level of these professionals, there are many who accept calling antidepressants “happy pills”. Well, they cause impotence: “How can men be happy if they become impotent and women, if they become frigid?”, asks Peter Gøtzsche.

The way forward is to invest in young people who will become psychiatrists. They will, I am sure, put an end to this matter of professionals who today, instead of caring for their psychiatric patients, turn them into victims. It will be hard work, but it needs to be done.

In this book, Peter Gøtzsche proposes ways to become free from these two present-day plagues of psychiatrists and psychotropic drugs. The following are some of these ways:

- Psychiatrists need to be reeducated so that they can function as psychologists.
- The focus should be on taking patients off psychiatric medications, given that they are harmful over the long term.
- A national network of 24-hour assistance and an associated website should be established to provide advice to people who have been harmed through dependence and have been taken off their prescribed drugs.
- “Say sorry”. It is very important that victims of psychotropic drug abuse should receive apologies. People in government need to demand that psychiatric associations should apologize for the harm that has been caused to patients and for the systematic lies that have been told regarding protection through these drugs, against suicide or brain damage.
- Psychiatric diagnostic systems such as DSM-5 and CID-11 should be completely discarded. (My note: this is perhaps the most important step of all, given that these two catalogues not only are extremely poorly produced and worthless, but also have become “bibles” and the only “scientific” source for decadent psychiatry.)
- The psychiatric medications available should only be taken under strictly controlled circumstances.
- Nobody who works with psychiatric patients should have financial conflicts of interest with the pharmaceutical companies.
- All of us should do what we can to change the deceitful narrative of psychiatry.

I am sure that everyone who reads the book “Mental health survival kit”^1^ will see both the size of the problem and some solutions that are feasible.

## References

[1] Gøtzsche PC (2020). Mental health survival kit and withdrawal from psychiatric drugs.

